# Biodegradable Rice Starch/Carboxymethyl Chitosan Films with Added Propolis Extract for Potential Use as Active Food Packaging

**DOI:** 10.3390/polym10090954

**Published:** 2018-08-28

**Authors:** Rungsiri Suriyatem, Rafael A. Auras, Chitsiri Rachtanapun, Pornchai Rachtanapun

**Affiliations:** 1 Division of Food Science and Technology, Faculty of Agro-Industry, Chiang Mai University, Chiang Mai 50100, Thailand; suriyatem.k@gmail.com; 2 School of Packaging, Michigan State University, East Lansing, MI 48824, USA; aurasraf@msu.edu; 3 Department of Food Science and Technology, Faculty of Agro-Industry, Kasetsart University, Bangkok 10900, Thailand; chitsiri.t@ku.ac.th; 4 Division of Packaging Technology, Faculty of Agro-Industry, Chiang Mai University, Chiang Mai 50100, Thailand

**Keywords:** antimicrobial, antioxidant, bio-based films, compostability, propolis, swelling

## Abstract

Active films from rice starch/carboxymethyl chitosan (RS/CMCh) incorporated with propolis extract (ppl) were developed and characterized. The effect of the ppl content (0–10% w/w based on RS/CMCh) on the developed films’ properties were determined by measuring the optical, mechanical, thermal, swelling, barrier, antimicrobial, and antioxidant attributes. The thermal stability and biodegradability of the films were also investigated. As the ppl content increased, free radical scavenging and a* and b* color values increased, whereas luminosity (L*) and swellability of the films decreased. The active films with 5–10% ppl possessed antimicrobial ability against Gram-positive bacteria (*Staphylococcus aureus* and *Bacillus cereus*). The active film with 10% ppl displayed increased flexibility and thermal stability, without a change in oxygen permeability. The results indicated that incorporation of ppl into RS/CMCh film could enhance the films’ antioxidant and antimicrobial properties.

## 1. Introduction

Biodegradable and bio-based packaging materials are of growing interest, due to food safety and environmental problems caused by the use of fossil plastic and the generation of white pollution. Polymers derived from some polysaccharides—such as starch, cellulose derivatives, and chitosan—are edible and biodegradable [1]. Polysaccharide films are generally efficient gas barriers, with good optical properties and moderately good mechanical properties at low relative humidity (RH) [2]. In addition, biodegradable polysaccharide films can be modified with functional compounds to create active food packaging films [1,3,4,5,6]. Antioxidant and antimicrobial agents, which may be migrating from the packaging to the food products or surrounding headspace, have been used to extend the shelf life of food and improve its quality [6]. Antioxidant films could be used to protect fatty food against oxidative damage [6].

Bee products—such as honey, bee pollen, and propolis—have been found to possess antioxidant and antimicrobial activities [7,8]. Propolis is stated to have better radical scavenging capability than other bee products [7,9,10]. It is a natural resinous substance collected by honeybees [9] and contains polyphenols, such as flavonoids and phenolic compounds, which are the most important active ingredients responsible for its biological activity [3,11]. Antimicrobial effects of propolis against Gram-positive and Gram-negative bacteria and antioxidant activities have been reported [9,10,12]. However, few studies have documented the addition of propolis into biopolymer films, such as chitosan [3] and cassava starch films [4,5], to investigate the effect of propolis on the films’ properties and functionality.

We have previously demonstrated that the blending of rice starch (RS) and carboxymethyl chitosan (CMCh) improved the strength, flexibility, thermal stability, and biodegradation of compostable blend RS/CMCh films [13]. Those results indicated that RS/CMCh blended films have the potential to be used as biodegradable, edible films. We have also identified that the optimum content of RS/CMCh leading to a strong, flexible and durable film was 50:50 w/w. Hence, this work aimed to create active packaging films by adding propolis to RS/CMCh 1:1-based active packaging films.

RS/CMCh 50:50 films were produced and modified by blending with propolis extract (ppl; 0, 2.5, 5 and 10% w/w, based on RS/CMCh). Films were characterized by color, opacity, morphology, X-ray diffraction (XRD) and Fourier transform infrared (FTIR) spectroscopy, thermal and mechanical properties, oxygen and water barrier, swelling, and biodegradation. The influence of ppl on the total phenol content (TPC) and antimicrobial and antioxidant activities of the developed films were also evaluated.

## 2. Materials and Methods

### 2.1. Materials and Microorganisms

Native RS (Rose 100R; Thai Flour Industry Company Ltd., Bangkok, Thailand), shrimp chitosan with 98% degree of deacetylation and 900,000–1,300,000 Da molecular weight (Taming Enterprises, Samut Sakhon, Thailand), glycerol (Union Science Co., Ltd., Chiang Mai, Thailand) and dried ppl extract, collected from Chiang Mai Province (T. Man Pharma Co., Ltd., Bangkok, Thailand) were used for the preparation of the film-forming solution. All reagents were of analytical grade and used as received. Nutrient agar was purchased from HiMedia Laboratories Pvt., Ltd. (Mumbai, Maharashtra, India). NaCl was from QRёC (Auckland, New Zealand).

*Staphylococcus aureus* (TISTR 517), *Bacillus cereus* (TISTR 687), and *Escherichia coli* (TISTR 1261) were provided by the Division of Biotechnology, Faculty of Agro-Industry, Chiang Mai University (Chiang Mai, Thailand).

### 2.2. Preparation of RS/CMCh/ppl Films

Before the film preparation, chitosan was converted to CMCh (degree of substitution = 0.49), as previously described by the authors [13]. RS/CMCh 3% (w/v) polymer solution in a ratio of 50:50 with glycerol at 25% (w/w, based on RS/CMCh content) as a plasticizer was prepared by following the method described by Suriyatem et al. [13]. Different amounts of ppl (0, 2.5, 5.0 and 10.0% w/w, based on RS/CMCh content) were added to the polymer solution heated at ~45 °C. The mixture was then constantly stirred by a magnetic stirrer (IKA C-MAG HS7, Wilmington, NC, USA) for 15 min, cooled to 25 °C and degassed, and 130 mL of the mixture was cast onto an acrylic casting plate (0.15 × 0.15 m) at 25 °C for 36 h. The dried films were peeled and stored in a sealed aluminum bag until further characterization. The thickness of the films was measured by using a GT-313-A micrometer (Gotech Testing Machine Inc., Taichung, Taiwan). The film density was determined by weighing the film and recording its volume, according to the method described by Sun et al. [1]. Table 1 lists the sample codes and composition, the film thickness and density.

### 2.3. Characterization

A Jeol JSM-5910LV scanning electron microscope (JEOL USA Inc., Peabody, MA, USA) was used to observe the cross-sectional surface morphology of the films. The films were cut in liquid nitrogen using a sharp razor blade and adhered on specimen stub with carbon tape. The films were imaged at an accelerating voltage of 15 kV with gold coating using a sputter coater (SPI-Module, West Chester, PA, USA).

Tensile strength (*TS*), elongation at break (*EB*), and modulus of elasticity (*E*) of five film specimens (0.025 × 0.100 m) were measured by using a universal testing machine (United Calibration Corp., Huntington Beach, CA, USA) with a 5 kN load cell equipped with tensile grips, according to ASTM D882-12 [14] and the method specified by Suriyatem et al. [13]. The initial grip separation and cross-head speed were set at 50 mm and 50 mm/min, respectively.

FTIR analysis was run by using an FTIR spectrometer (Equinox 55, Bruker, Billerica, MA, USA), to observe the structural interfaces of film samples. The film was cut to fit and placed in the sample holder. The spectra were recorded over the wavenumber range 4000–400 cm^−1^ at a resolution of 4 cm^−1^.

The crystal structures of the produced films were determined by an X-ray diffractometer (MiniFlex II, Rigaku, Japan) using Cu Kα radiation at a voltage of 30 kV and 15 mA with a scattering angle (2θ) from 5 to 60° at a scan speed of 5°/min.

The swelling ratio of the films was tested according to a previously described method [13]. Briefly, duplicate film samples (0.02 × 0.02 m) were immersed in distilled water at 23 °C. Weight gain of the swollen films was measured at a specific time, and the swelling ratio was calculated using Equation (1).

(1) $$\mathrm{Swelling}\;\mathrm{ratio}\;=\frac{W_{2}-W_{1}\;}{W_{1}}\times 100,$$

where *W*_1_ and *W*_2_ are the weight of the film samples before and after immersion in water, respectively.

A Q100 differential scanning calorimetry (DSC) apparatus (TA Instruments, New Castle, DE, USA) was used to examine the films’ thermal properties. Each film sample (5–10 mg) was placed in an aluminum pan. The aluminum pans were hermetically encapsulated and measured using a heat–cool–heat cycle. The test was run between −50 to 250 °C at a scanning rate of 10 °C/min under an N_2_ atmosphere (70 mL/min). The test was run in duplicate.

Thermogravimetric analysis (TGA) was carried out using a TGA Q50 instrument (TA Instruments). Pieces of 5–10 mg of each film were heated from 30 to 600 °C at 10 °C/min, under a dry N_2_ atmosphere (70 mL/min). The test was run in duplicate.

The color of the films was determined using a colorimeter (CR-10, Konica Minolta, Osaka, Japan), to record the L* (lightness), a* (redness/greenness), and b* (yellowness/blueness) values. The type of illuminant was D65 and illuminating/viewing geometry was 8°/d (8 degree illumination diffuse reflectance reception). Opacity (*Op*) was evaluated by measuring the absorbance at 550 nm (A550) of the films using a Spectro SC spectrophotometer (LaboMed, Inc., Los Angeles, CA, USA) [13]. The film sample was placed into the spectrophotometer holder and the absorbance was recorded. The *Op* was calculated as

*Op* = A550/*x*, (2)

where *x* is the film thickness (mm). All measurements were run in triplicate.

Water vapor permeability (WVP) of the films was performed according to ASTM E96/E96M-16 [15] as described by Suriyatem et al. [13]. Briefly, a test cup containing dried silica gel was covered with a film sample and sealed with paraffin wax. The sample cup was placed at 65% RH, 25 °C. The weight of the sample cup was recorded every day, and the WVP was calculated [13]. All measurements were run in triplicate.

Oxygen permeability (O_2_P) of the films was measured at 23 °C, 50% RH and 100% O_2_ by an 8001 oxygen permeation analyzer (Illinois Instruments, Inc., Johnsburg, IL, USA) in accordance with ASTM D3985-05 [16], as described by Suriyatem et al. [13]. Briefly, duplicate film samples were placed on a sample chamber with an expose testing area of 3.14 cm^2^. The test was run continuously until a steady-state was reached. The O_2_P calculation method is also provided elsewhere [13].

### 2.4. TPC

The TPC of the films was determined using the Folin–Ciocalteu method, as described by Suriyatem et al. [9] with slight modifications. Briefly, a film sample (0.02 × 0.02 m) was soaked in 10 mL of absolute methanol for 24 h, to prepare a film extract solution containing phenolic compounds. The extract solution (0.3 mL), distilled water (3 mL), 2.0 N Folin–Ciocalteu reagent (0.25 mL) and 2.5 mL of 7% (w/v) sodium carbonate were added and mixed in a test-tube. The tube was incubated in the dark for 30 min, and the absorbance was spectrophotometrically measured at 760 nm using a Spectro SC spectrophotometer (LaboMed, Inc., Los Angeles, CA, USA). The results were expressed as milligrams of gallic acid equivalents (GAE) per gram of sample [9].

### 2.5. Antioxidant Activity

The in vitro antioxidant activity of the films was monitored by the 2,2-diphenyl-1-picrylhydrazyl (DPPH) assay, according to the method described by Suriyatem et al. [9] with slight modification. In brief, the film extract solution (1 mL), which was prepared as described above in the TPC determination, was mixed with 0.06 mM DPPH–methanol solution (2 mL). The mixture was placed in a dark room for 30 min. The spectrophotometric absorbance was determined at 516 nm. The activity was given as %DPPH inhibition, which was calculated as described elsewhere [9].

### 2.6. Antimicrobial Capacity

Antimicrobial capacity of the film was determined by using the agar disc diffusion assay, following the method of Jutaporn et al. [17] with slight modification. The inocula (*S. aureus*, *B. cereus*, and *E. coli*) were prepared as described elsewhere [9]. The final concentration of the bacterial cell numbers (~10^5^–10^6^ CFU/mL) was obtained by diluting with sterile NaCl solution. The film disc was aseptically cut into ϕ = 6 mm and placed on a nutrient agar plate. The medium had been previous seeded with the test bacteria (100 μL). The plate was incubated at 30 °C for 24 h. The diameter of inhibition zone was measured. The tests were performed in triplicate.

### 2.7. Biodegradability

The biodegradability of the films was determined in simulated compost environmental conditions, as previously described by some of the authors [13,18] and according to ASTM D5338-15 [19]. The test was operated by using an in-house built direct measurement respirometric (DMR) system [20]. Briefly, the film sample was cut into pieces of around 0.01 × 0.01 m before testing. The sample (8 g) and compost (400 g) were mixed thoroughly and loaded in a glass jar bioreactor. The bioreactor was incubated in a dark place for 87 days, at 58 ± 2 °C, 50 ± 10% RH, with an air flow rate of 40 sccm^3^/min. Cellulose powder was used as a positive control, and a blank bioreactor was the bioreactor containing only compost. The amount of CO_2_ released from the reactor was recorded as a function of time. The %mineralization was calculated as described elsewhere [20].

### 2.8. Statistical Analysis

Data were analyzed by one-way analysis of variance (ANOVA) and Duncan’s multiple range tests (*p* ≤ 0.05) using SPSS software (version 11, SPSS Inc., Chicago, IL, USA).

## 3. Results and Discussion

### 3.1. Microscope Observations

**Figure 1:**
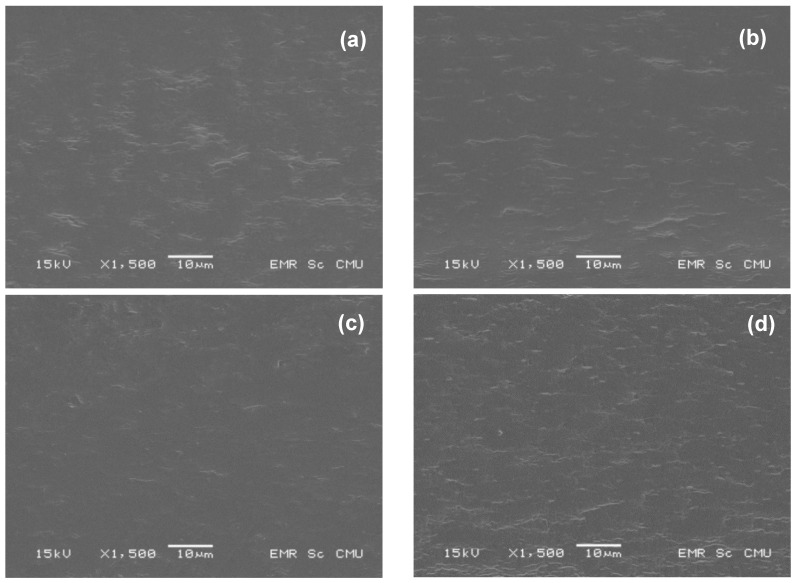
SEM images of (**a**) RS/CMCh film (control) and RS/CMCh film with (**b**) 2.5% ppl (**c**) 5.0% ppl and (**d**) 10.0% ppl.

Developed films with thickness of around 0.15–0.16 mm were obtained (Table 1). Figure 1 shows the scanning electron microscopy (SEM) images of the fractured surface of control (a) and active films (b–d). The control film displayed a homogenous structure, suggesting the high miscibility between the components. The active films also showed a homogenous structure with a rough surface. The fractured surface of ppl-10.0% seemed to be rougher than the other films.

### 3.2. Mechanical Properties

**Figure 2:**
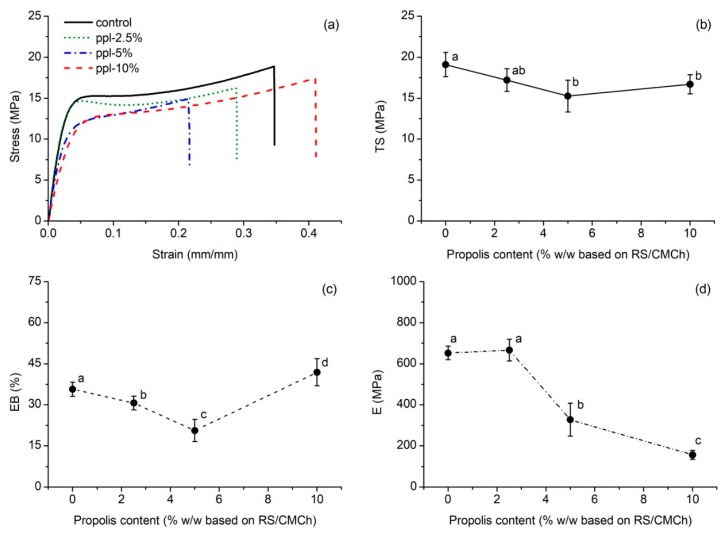
Tensile properties of the developed films; (**a**) stress–strain plot, (**b**) *TS*, (**c**) *EB*, and (**d**) *E* values as a function of propolis content. Values with the same lowercase letter (above a point) are not significantly difference (*p* > 0.05).

Figure 2a presents an example of the stress-strain curve for the produced films. The effects of the ppl quantity on the mechanical properties of RS/CMCh-based films are shown in Figure 2b–d. The result showed that as ppl content increased (0–5%), *TS* decreased from 19.1 to 15.3 MPa (Figure 2b). However, the *TS* of the films incorporated with 5% and 10% ppl were not significantly different from each other (*p >* 0.05). The *EB* decreased from 35.7 to 20.6% with an increase of ppl from 0 to 5%, and then it increased to 41.9% with 10% ppl (Figure 2c) while *E* tended to decrease as the ppl content increased (Figure 2d). The network microstructure, intermolecular forces, and crystallinity of the film play an important role in its mechanical properties [1]. The incorporation of polyphenols into the polymer films is reported to decrease the mechanical properties [1,21,22]. In this work, polyphenols in ppl may hinder the formation of an ordered crystalline structure in the RS/CMCh matrix, weaken the intermolecular hydrogen bonding, and interrupt the interaction between polymer–polymer chains, including polymer–glycerol interactions. These phenomena will be further discussed in Section 3.3 and Section 3.4. The increased *EB* at 10% ppl implied a more flexibility film than the others, and it was also supported by the SEM image. At this level, ppl may show a plasticizing effect.

### 3.3. FTIR

**Figure 3:**
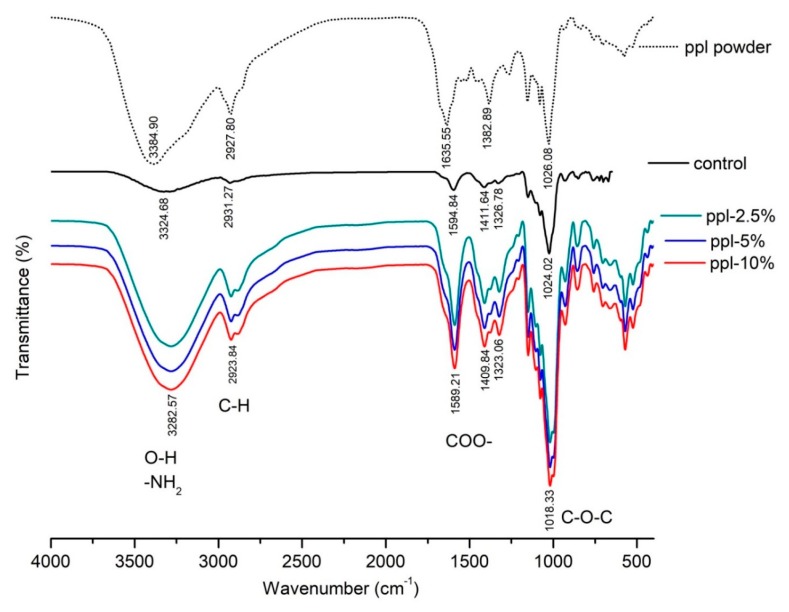
FTIR spectra of the ppl powder and the produced films.

Intermolecular interaction between RS/CMCh and ppl was investigated by FTIR analysis. Figure 3 presents the FTIR spectra of ppl powder, RS/CMCh (control) film and RS/CMCh/ppl (active) films. The spectra of control film showed major bands at 3324.68, 2931.27, 1594.84, and 1024.02 cm^−1^, corresponding to the O–H and N–H (NH_2_) stretching, C–H stretching (CH_2_), asymmetric COO– stretching and C–O stretching vibration, respectively [13]. The ppl powder spectrum showed a shoulder in the band at 1635.55 cm^−1^, which was not visible in the control spectrum but was in films with ppl. This band was corresponding to C=C ring skeletal stretching vibration of the aromatic group [23]. All films, with and without ppl, showed a similar pattern in the FTIR spectra. However, specific peaks of all active films were shifted to lower wavenumbers than the control films, indicating weak intermolecular interactions of the developed films [24]. The transmittance intensities of each active film were dramatically higher than the control film. These observations confirmed the presence of a phenolic compound of ppl in the films. Kaewmanee [25] noted that propolis collected from Chiang Mai, Thailand, is composed of a variety of phenolic compounds, such as 37% pinostrobin, 7% quercetin, 6% pinocembrin, and 6% caffeic acid. These phenolic compounds have a benzene ring, –OH or –CH_3_ group, C=O, and C–O in their structure [25]. However, there were no significant differences between the spectra of the group of active films. The interaction between ppl and the polymer RS/CMCh seemed to be more likely to be physical. Besides, the interaction between ppl and CMCh may occur via their –OH and COO– groups, respectively. This interaction may compete to obstruct the formation of intermolecular hydrogen bonds between RS and CMCh.

### 3.4. XRD

**Figure 4:**
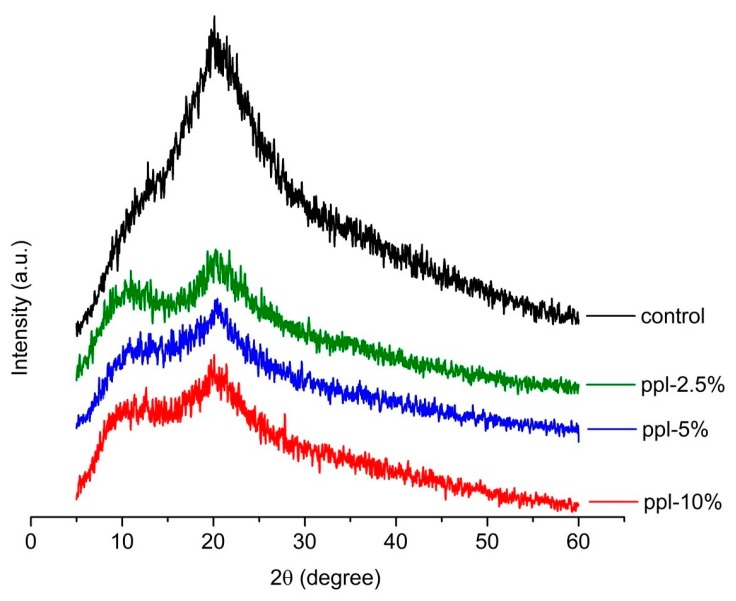
XRD pattern of the RS/CMCh/ppl blended films.

Figure 4 displays the XRD spectra of the developed films. The XRD analysis was run to additionally observe the effect of ppl on the crystalline structures of the RS/CMCh-based films. The spectra revealed two main broad diffraction peaks at around 11.9 and 20.0°, respectively, in the control film [13]. After 2.5–10.0% ppl was incorporated into the films, the diffraction peaks at 21° still existed but became flattened. This observation is due to reduced crystallinity in the developed films. Therefore, ppl disrupts the crystallization process of the RS/CMCh blended films. The interaction between ppl and RS/CMCh matrix may cause the competitive effect to disrupt the strong intermolecular hydrogen bonds between RS and CMCh, resulting in decreased crystallinity [1] and lowering of the active films’ mechanical properties (Figure 2), as described above.

### 3.5. Swelling

**Figure 5:**
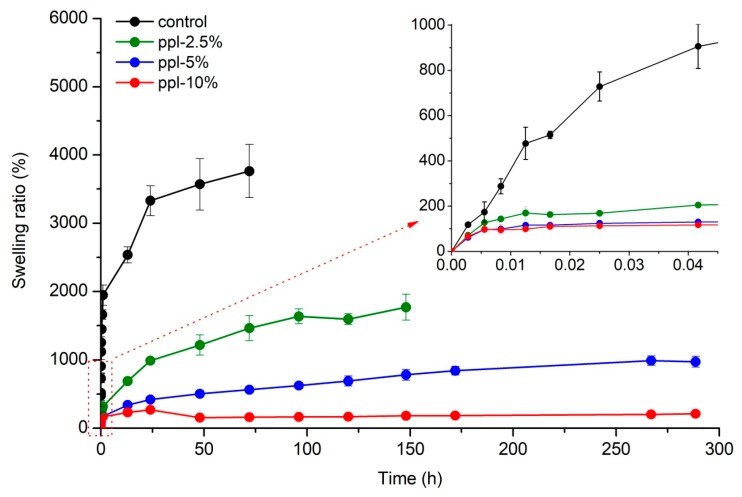
Swelling properties of the developed films.

Figure 5 shows the swelling ratio of RS/CMCh/ppl blended films increased with an increase of time. The test was stopped when the equilibrium swelling ratio (ESR) was achieved. Control and ppl-2.5% films began to crack and disintegrate with water at 72 and 148 h, respectively, so their test was stopped at those times. The ESR of the developed films gradually decreased from 3764 to 211% when the ppl amount increased from 0 to 10%. The decrease in swelling capacity when adding ppl could be attributed to a reduction in the strong hydrogen bonds between RS and CMCh and an increase in hydrophobic groups (aromatic rings) of the polyphenols in ppl, as more ppl was introduced. A similar observation was reported by Aadil et al. [22] for lignin/gelatin films, whereby the swelling percentage of the films decreased with an increase in the lignin/gelatin ratio [22].

### 3.6. Thermal Properties

**Figure 6:**
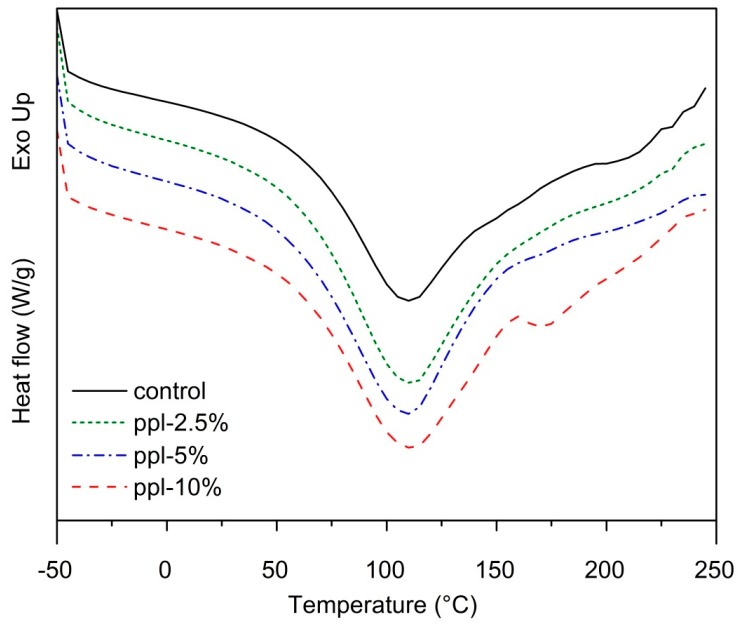
DSC thermographs of the developed films.

DSC analysis of the RS/CMCh/ppl films demonstrated an endothermic peak at 108–111 °C (*p >* 0.05) in the thermograms of each film, which was attributed to the evaporation of water (Figure 6). However, at 10% ppl, the blended film exhibited a small peak at around 170 °C, possibly due to the decomposition of ppl, because propolis can decompose at above 150 °C [26].

**Figure 7:**
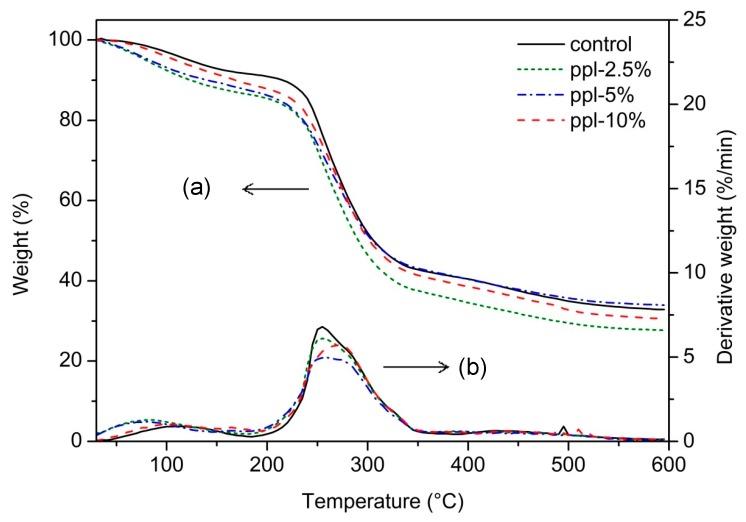
Thermal stabilities of RS, CMCh, and RS/CMCh blend films; (**a**) TGA thermogram and (**b**) DTG thermogram.

Thermal stability of the RS/CMCh/ppl films was analyzed using TGA (Figure 7). To determine the thermal decomposition temperatures (*T_d_*) of each film, the first derivative function of each TGA curve was deduced. The results showed that the *T_d_* of the control and ppl-2.5% films occurred in two main steps. The first step (104 °C) corresponded to water loss, and the second step (254 °C) denoted degradation of the components of RS and CMCh. In contrast, the blended films carrying 5–10% ppl showed three main steps of the *T_d_*: water loss (90–94 °C), degradation of propolis (162–167 °C), and RS/CMCh decomposition (257–274 °C). The peak of glycerol decomposition was not found for each film. At 2.5–5.0%, the ppl did not influence the *T_d_* of the RS and CMCh components of the active films (*p >* 0.05). However, 10% ppl increased the *T_d_* from 255 °C (control) to 274 °C (*p* < 0.05). When added in this proportion, the active film showed enhanced thermal stability property. The final weight loss (68–74%) was not significantly different among the films, (*p >* 0.05), corroborating the non-significant differences in their carbon contents, which was in the range 36.6–37.6% (*p >* 0.05).

### 3.7. Color and Op

Color and *Op* of a product affect its consumer acceptance. Table 2 lists the L*, a*, b*, and *Op* of the films. All color parameters were observed to be affected by the ppl quantity (0–10%). The control film had a higher L* and lower a* and b* values than the active films. When the ppl content increased, L* significantly decreased (*p* < 0.05), and a* and b* significantly increased (*p* < 0.05). Sun et al. [1] reported a similar tendency for each color parameter in chitosan films incorporated with various amounts of thinned young apple polyphenols. Our results indicated that ppl tended to enhance redness and yellowness and diminish lightness of the active films. These color changes may be attributed to the effect of original yellow pigmentation of ppl. However, the *Op* of the active films was not significantly different compared to the control film (*p >* 0.05). The results revealed that the RS/CMCh/ppl films might be able to protect the products from visible and ultraviolet light, which can contribute to the loss of nutrients and discoloration of food products [1].

### 3.8. WVP and O_2_P

Table 2 shows the WVP values of the control and active films. RS/CMCh with 2.5% and 5.0% ppl showed similar WVP values (*p >* 0.05), which were comparatively lower than the 10% ppl films (*p* < 0.05). This result was probably attributed to the plasticizing effect of ppl at a high level of incorporation (10%). The WVP of films is also a balance of the hydrophilic/hydrophobic ratio of the film components [6]. The presence of –OH and COO– functional groups was responsible for the WVP of the RS/CMCh/ppl blended films. Besides, water barrier properties may be related to the density of the films [1]. The ppl-10.0% film had a lower density (*p* < 0.05) than the others (Table 1) so water vapor molecules were able to permeate the film more easily. Similar findings were documented by Han et al. [27] and Piñeros-Hernandez et al. [6], who investigated sodium alginate/carboxymethyl cellulose/cinnamon oil films and cassava starch/rosemary extract films, respectively. Although the addition of 10% ppl increased the WVP of the RS/CMCh/ppl films, the value was still lower than those of some biopolymer-based films, such as chitosan/thinned young apple polyphenols films (7.5–10.7 × 10^−14^ kg·m/Pa·m^2^·s) [1], cassava starch/rosemary extract films (5.8–11.0 × 10^−13^ kg·m/Pa·m^2^·s) [6], sodium alginate/carboxymethyl cellulose/cinnamon oil films (1.4–2.9 × 10^−12^ kg·m/Pa·m^2^·s) [27], and chitosan/propolis films (5.8–6.4 × 10^−12^ kg·m/Pa·m^2^·s) [3].

Table 2 also shows the O_2_P values of the RS/CMCh/ppl films. The data suggested that when 2.5–5.0% ppl was incorporated into the films, the O_2_P values did not change significantly (*p* > 0.05). However, the O_2_P value slightly increased when 10.0% ppl was employed when compared with the 2.5–5.0% ppl, but it was not significantly different than the control film (*p* > 0.05). This slight increase in the O_2_P value may also be due to increased O_2_ solubility in the hydrophobic part of ppl, which facilitated O_2_ transmission through the film. Nevertheless, our films still had lower O_2_P values relative to a methylcellulose film (30.2 × 10^−19^ kg·m/Pa·m^2^·s) [2] and much lower than some synthetic polymer-based films, such as polyethylene (64–281 × 10^−19^ kg·m/Pa·m^2^·s) [28] and low-density polyethylene/polyaniline blends (114–163 × 10^−19^ kg·m/Pa·m^2^·s) [29].

### 3.9. TPC

The Folin–Ciocalteu phenol reagent was employed to obtain an estimate of the phenolic content in the RS/CMCh/ppl films. The TPC in the films significantly increased (*p* < 0.05) when the ppl content was increased (Table 2). This trend was also seen for other biopolymer films, including chitosan/green tea extract films [30] and cassava starch/propolis films [5].

### 3.10. Antioxidant Activity

The DPPH assay was used to indicate the in vitro antioxidant activity of the films. It was seen that %DPPH inhibition of the films increased with increasing ppl content and TPC value (Table 2). The antioxidant activity of the products was related to the TPC value [7,9,11,12]. The film without propolis also showed some DPPH radical scavenging activity but it was less than that of the active films (*p* < 0.05). This result may be attributed to the interaction of the free DPPH radicals with the residual free NH_2_ groups of the CMCh in the RS/CMCh film. NH^3+^ groups could be formed from NH_2_ groups and hydrogen ion in the solution [30]. Siripatrawan and Harte [30] found similar results in chitosan/green tea extract films.

### 3.11. Antimicrobial Ability

Table 3 reveals the RS/CMCh film without ppl and with 2.5% ppl did not show any inhibition zone with the tested bacteria. When 5% and 10% ppl were introduced, antimicrobial activity of the films was evident for *S. aureus* and *B. cereus.* The antimicrobial ability depends on the polarity and compatibility between the antimicrobial agent and the polymer [31]. Therefore, the data indicate good compatibility of the polymer matrix with ppl, which was supported by the SEM observation. Propolis has a high TPC (237 mg GAE/g of sample) compared to honey and bee pollen and causes inhibition and lethality of *S. aureus*, *B. cereus*, and *E. coli* [9]. The antimicrobial activity of the films was likely ascribed to the phenolic compounds contained in ppl, such as flavonoids. However, no inhibition zone for *E. coli* was observed in all the films. This study revealed that the active films inhibited the Gram-positive bacteria (*S. aureus* and *B. cereus*), but not the Gram-negative (*E. coli*) at these ppl levels investigated, which may be because of the structural differences in the bacterial cell wall. Gram-negative bacteria have an additional outer layer membrane composed of lipopolysaccharides (endotoxins), lipoprotein, and phospholipids when compared with Gram-positive bacteria that allows for a more effective action against foreign molecules [32]. This observation concurs with previous studies of antimicrobial activity of chitosan–propolis films [3] and chitosan–young apple polyphenol films [1] against Gram-positive and Gram-negative bacteria.

### 3.12. Biodegradability

**Figure 8:**
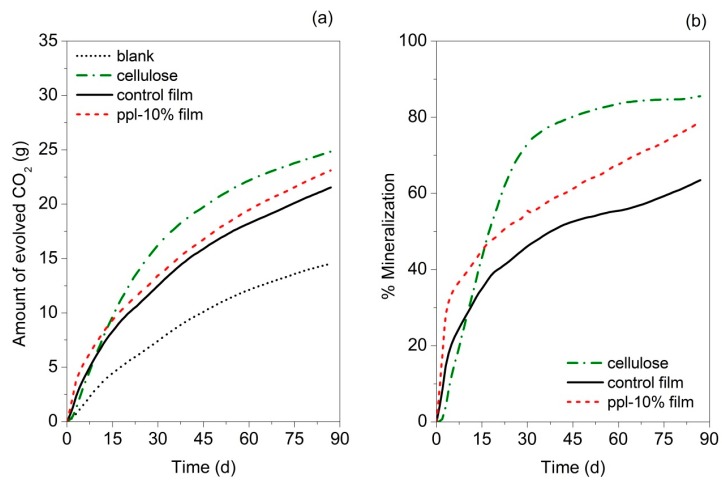
(**a**) Amount of evolved CO_2_ of control and active films, cellulose and blank compost; (**b**) %Mineralization of control and active films and cellulose, at 58 ± 2 °C and approximately 50 ± 10% RH in yard waste compost.

The biodegradability of the control and ppl-10.0% films in simulated compost environmental conditions was investigated using the in-house built DMR system. The ppl-10.0% film was selected for this test due to its good mechanical properties (comparative strength and higher flexibility) and better antioxidant and antibacterial activities among RS/CMCh/ppl films. Figure 8a shows the CO_2_ evolution from the films during the 87-day test period. Cellulose was used as a positive control. At the end of the test, the amount of cumulative CO_2_ of the 10.0% ppl–carrying film was higher than the control film but lower than cellulose. Figure 8b presents the %mineralization of the developed films. %Mineralization is the %carbon molecules present in the sample converted to CO_2_ during the test when compared to the total available carbon [20]. It was demonstrated that ppl-10.0% film has a higher biodegradability than the control film. The faster degradation of ppl-10.0% is likely due to its relatively higher available amount of OH groups. The increase in the presence of OH groups was previously found to increase the degradation rate of gelatin/carboxymethyl cellulose/chitosan films [33]. This observation supported the evidence found in the FTIR assay. Jaramillo et al. [34] also noted that decomposition of cassava starch films is faster with the addition of yerba mate extract while Piñeros-Hernandez et al. [6] recorded that biodegradation of cassava starch films was retarded by the presence of rosemary extract (RE). A reduction in the OH amount of the cassava starch/RE film was found when rosemary extract was introduced that might be responsible for the biodegradation inhibition [6].

At 87 days, the %mineralization of ppl-10.0% film was 92.2% of the cellulose (positive control). It indicated that ppl-10.0% could be defined as passing the biodegradability requirement, based on ASTM D6400-12 [35].

## 4. Conclusions

RS/CMCh blended films carrying propolis extracts showed antioxidant activity and antimicrobial properties against Gram-positive microorganisms. When 10% of the extract was incorporated, the active film possessed maximum flexibility, thermal stability, DPPH radical scavenging activity, and TPC. The results showed that the developed active film has the potential to be used as an active food packaging film. The improved biodegradability of the active film as compared to the control also supported the advantages of the film when disposed of in a composting environment.

## Figures and Tables

**Table 1:** Code, composition, thickness, and density of RS/CMCh/ppl films.

Film Code	ppl (% w/w *)	Film Thickness (mm)	Film Density (g/cm^3^)
Control	0.0	0.147 ± 0.003 ^a^	1.118 ± 0.003 ^a^
ppl-2.5%	2.5	0.148 ± 0.002 ^a^	1.129 ± 0.018 ^a^
ppl-5.0%	5.0	0.146 ± 0.002 ^a^	1.168 ± 0.010 ^b^
ppl-10.0%	10.0	0.162 ± 0.005 ^b^	1.046 ± 0.005 ^c^

Values in the same column with different letters are statistically significantly different (*p* ≤ 0.05). * Based on rice starch/carboxymethyl chitosan (RS/CMCh) content.

**Table 2:** Color (L*, a*, b*), opacity (*Op*), oxygen permeability (O_2_P), water vapor permeability (WVP), total phenol content (TPC), and DPPH antioxidant activity of the RS/CMCh/ppl blended films.

Film	L*	a*	b*	*Op*	O_2_P (×10^−19^ kg·m/Pa·m^2^·s)	WVP (×10^−14^ kg·m/Pa·m^2^·s)	TPC (mg·GAE/g·Sample)	DPPH (%·Inhibition)
Control	96.07 ± 0.49 ^a^	–1.12 ± 0.09 ^a^	06.62 ± 0.47 ^a^	1.71 ± 0.35 ^ab^	5.00 ± 0.51 ^ab^	4.66 ± 0.30 ^a^	-	39.3 ± 1.6 ^a^
ppl-2.5%	84.15 ± 0.50 ^b^	–0.22 ± 0.18 ^b^	30.08 ± 0.72 ^b^	2.31 ± 0.54 ^ab^	4.78 ± 0.04 ^a^	4.82 ± 0.81 ^a^	0.210 ± 0.004 ^a^	45.7 ± 1.6 ^b^
ppl-5.0%	81.04 ± 0.84 ^c^	0.80 ± 0.36	38.57 ± 0.85 ^c^	1.63 ± 0.19 ^a^	4.73 ± 0.08 ^a^	4.36 ± 0.53 ^a^	0.244 ± 0.002 ^b^	55.0 ± 0.8 ^c^
ppl-10.0%	74.08 ± 0.92 ^d^	5.78 ± 0.43 ^d^	49.38 ± 0.55 ^d^	2.04 ± 0.11 ^b^	5.74 ± 0.18 ^b^	5.86 ± 0.23 ^b^	0.408 ± 0.001 ^c^	69.9 ± 3.3 ^d^

Notes: Values area reported as average ± standard deviation. Values in the same column with different letters are statistically significantly different (*p* ≤ 0.05).

**Table 3:** Antibacterial activity of different rice starch/CMCh/ppl blended films.

Film	Inhibition Zone (cm)		
	*Staphylococcus aureus*	*Bacillus cereus*	*Escherichia coli*
Control	N.D.	N.D.	N.D.
ppl-2.5%	N.D.	N.D.	N.D.
ppl-5.0%	0.81 ± 0.08 ^a^	0.73 ± 0.03 ^a^	N.D.
ppl-10.0%	0.82 ± 0.12 ^a^	1.02 ± 0.04 ^b^	N.D.

Values in the same column with different alphabetic symbol are statistically significantly different (*p* ≤ 0.05). Diameter of the film samples = 0.6 cm. N.D. = not detected.
